# Depressive Symptoms and Mortality Among US Adults

**DOI:** 10.1001/jamanetworkopen.2023.37011

**Published:** 2023-10-09

**Authors:** Zefeng Zhang, Sandra L. Jackson, Cathleen Gillespie, Robert Merritt, Quanhe Yang

**Affiliations:** 1 Division for Heart Disease and Stroke Prevention, US Centers for Disease Control and Prevention, Atlanta, Georgia

## Abstract

**Importance:**

Depression is a common mental health disorder in the US. Depressive symptoms have been associated with increased cardiovascular disease incidence and mortality, but studies have largely focused on narrow population subgroups.

**Objective:**

To examine the association between depressive symptoms and mortality in a large, diverse, nationally representative sample of US adults, and to examine how lifestyle factors mediate this association.

**Design, Setting, and Participants:**

This was a prospective cohort study of a nationally representative sample of US adults using National Health and Nutrition Examination Survey 2005 to 2018 data linked with the National Death Index through 2019 for adults aged 20 years and older. Data were analyzed between March 1 and May 26, 2023.

**Main Outcomes and Measures:**

All-cause, cardiovascular disease, and ischemic heart disease mortality. Depressive symptoms were defined by Patient Health Questionnaire-9 scores and were categorized as none or minimal, mild, and moderate to severe. Secondarily, we assessed degree of mediation by lifestyle factors.

**Results:**

A total of 23 694 participants were included (unweighted n = 11 862 male [weighted 49.8%]; mean [SE] age, 44.7 [0.24] years). Prevalences of mild and moderate to severe depression were 14.9% and 7.2%, respectively. For all-cause mortality, hazard ratios were 1.35 (95% CI, 1.07-1.72) for mild depressive symptoms vs none and 1.62 (95% CI, 1.24-2.12) for moderate to severe depressive symptoms vs none. The corresponding hazard ratios were 1.49 (95% CI, 1.11-2.00) and 1.79 (95% CI, 1.22-2.62) for cardiovascular disease mortality and 0.96 (95% CI, 0.58-1.60) and 2.21 (95% CI, 1.24-3.91) for ischemic heart disease mortality. The associations were largely consistent across subgroups. Approximately 11.0% to 16.1% of the associations between depression and mortality could be explained by lifestyle factors. Feeling tired or having little energy, poor appetite or overeating, and having little interest in doing things were independently associated with all-cause and cardiovascular disease mortality but not with ischemic heart disease mortality.

**Conclusions and Relevance:**

In this prospective cohort study of a nationally representative sample of US adults, there was a graded positive association between depressive symptoms and mortality. Public health efforts to improve awareness and treatment of depression and associated risk factors could support a comprehensive, nationwide strategy to reduce the burden of depression.

## Key Points

**Question:**

What is the association between depressive symptoms and death from all causes, cardiovascular disease, and ischemic heart disease?

**Findings:**

This cohort study of 23 694 individuals found a higher risk of all-cause, cardiovascular disease, and ischemic heart disease mortality among adults with moderate to severe depressive symptoms compared to those without depressive symptoms.

**Meaning:**

The findings of this study may help support the need for a comprehensive, nationwide strategy to reduce the burden of depression.

## Teaser

This cohort study evaluates the association between depressive symptoms and mortality among US adults.

## Introduction

Depression is a common mental health disorder among the US population. In 2020, an estimated 21 million US adults (8.4%) had at least 1 major depressive episode.^1^ Numerous adverse outcomes have been associated with depression, including increased cardiovascular disease (CVD) incidence and premature mortality.^2,3,4,5,6,7,8,9,10,11,12,13,14,15,16,17,18,19,20^ The onset of CVD occurs an average of 7.5 years earlier in adults with mood disorders,^21^ and people with severe mental disorders, including moderate to severe depression, die an average of 10 to 20 years earlier than the general population.^22^ In addition, depression is a significant determinant of quality of life and utilization of health services, accounting for approximately 50% of psychiatric consultations and 12% of all hospital admissions.^23^

Understanding the association between depressive symptoms and death from all-cause, CVD, and ischemic heart disease (IHD) may be an important step toward defining prevention and treatment strategies for both mood disorders and CVD. Previous studies of the associations between depression and incident CVD or mortality have mainly focused on middle-aged or older populations^3,9,14,15,20,24^ or select population subgroups.^2,4,25^ In addition, most previous studies analyzed the presence of depressive symptoms as a binary variable (eg, depressed or not)^2,4,7,8,9,10,13,14,15,16,18,19,20,24,25^ or used a total score that weighted each depressive symptom equally.^26^ Moreover, prior studies^16,18^ used the public-use mortality file for the National Health and Nutrition Examination Survey (NHANES) and could not fully capture CVD-related causes of death; they could not assess major cardiovascular disease overall nor IHD specifically. To our knowledge, there is no recent depression-mortality study among US adults using the NHANES restricted-use mortality data.

In this study, we used the 2005 to 2018 NHANES data linked to the National Death Index 2019, a prospective cohort of a nationally representative sample, to examine associations between depressive symptoms and all-cause, CVD, and IHD mortality, controlling for sociodemographic, lifestyle, and health status variables. We also examined the potential mediation of lifestyle factors, including alcohol consumption and the American Heart Association’s Life’s Essential-8 cardiovascular health metrics (LE-8),^27^ on the associations between depressive symptoms and mortality.

## Methods

### NHANES Study

NHANES uses a complex, stratified, multistage probability cluster sampling, cross-sectional design to collect health and nutritional data from a representative sample of the civilian noninstitutionalized US population. The design and operation of NHANES has been described previously.^28^ Study protocols for NHANES were approved by the National Center for Health Statistics Ethics Review Board. All adult participants provided signed informed consent. This study followed the Strengthening the Reporting of Observational Studies in Epidemiology (STROBE) reporting guidelines for cohort studies.

### NHANES 2005 to 2018–Linked Mortality (2005 to 2019)

To examine the association between depression and the risk of mortality, we used NHANES 2005 to 2018 Linked Mortality Files, linked to National Death Index death certificate records through 2019. For the linkage, both deterministic and probabilistic approaches were used to determine mortality status. Participant follow-up continued until death with censoring at the time of death for those who died from causes other than CVD or IHD. Participants not matched with a death record were considered alive through the entire follow-up period.^29^ The *International Classification of Diseases*, *10th Revision*, codes identified participants for whom major CVD (codes I00-I78) or IHD (codes I20-I25) was listed as the underlying cause of death. Linked mortality data are restricted; these data were accessed through the NCHS Research Data Center.

### Depressive Symptoms

Depressive symptoms were assessed using the Patient Health Questionnaire-9 (PHQ-9), a validated 9-item screening instrument that asks about the frequency of depressive symptoms over the past 2 weeks.^30^ Response categories of “not at all,” “several days,” “more than half the days,” and “nearly every day” receive a score of 0 to 3, respectively. Total PHQ-9 scores range from 0 to 27, with higher scores indicating more severe depression. PHQ-9 scores of 0 to 4, 5 to 9, 10 to 14, 15 to 19, and 20 to 27 represent none or minimal, mild, moderate, moderately severe, and severe depressive symptoms, respectively. Our analyses combined moderate, moderately severe, and severe depressive symptoms for the purpose of sample size. The PHQ-9 scores of 10 or greater had 88% sensitivity and 88% specificity for major depression,^30^ a well-validated cut point commonly used in clinical studies of depression.

### Covariates

Study covariates included age; sex; self-reported race and Hispanic ethnicity; education (<12, 12, or >12 years); marital status (never married, married/living with partner, or divorced/separated/widowed); poverty-to-income ratio (<1.30, 1.30-3.49, ≥3.50, and missing [n = 2009]); health insurance; family history of CVD; chronic kidney disease (albumin/creatinine ratio >30 mg/g or estimated glomerular filtration rate <60 mL/min per 1.73 m^2^); antidepressant use assessed from NHANES medication files; alcohol consumption (0, <2, or ≥2 drinks daily for men; 0, <1, or ≥1 drinks daily for women). Race and ethnicity were assessed as these variables are predictors of mortality and are associated with depression and other covariates.

The American Heart Association’s LE-8 includes body mass index (BMI, calculated as weight in kilograms divided by height in meters squared), nicotine exposure, physical activity, diet, sleep health, blood pressure, blood lipids, and blood glucose.^27^ Each LE-8 metric component score ranges from 0 to 100. The overall LE-8 score is the mean of 8 metric components and was categorized as high (≥80), moderate (50-79), and low (<50). The LE-8 definition and scoring methods are available in eTable 1 in Supplement 1.

### Statistical Analyses

Data were analyzed between March 1 and May 26, 2023. We estimated the prevalence of depressive symptoms in US adults and compared sociodemographic and clinical characteristics across depression categories (none or minimal, mild, and moderate to severe) using χ^2^ tests for categorical variables and analysis of variance for continuous variables.

Multivariable Cox proportional hazards models were used to estimate the hazard ratios (HRs) and 95% CIs of depression for all-cause, CVD, and IHD mortality, with none or minimal depression as the reference group. The base model adjusted for age, sex, race, and Hispanic ethnicity. The second model was additionally adjusted for education, marital status, poverty, health insurance, family history of CVD, chronic kidney disease, and antidepressant use. The third model was additionally adjusted for alcohol use and the LE-8 metrics. Interactions between depressive symptom categories and other sociodemographic and clinical characteristics were considered. We also examined associations between the 9 individual components of depressive symptoms and all-cause, CVD, and IHD mortality. The proportional hazards assumption of the Cox models was evaluated by the interactions of the predictors and log of survival time. Person-years of follow-up were calculated from the time of entry into the study until date of death or study termination. We calculated Kaplan-Meier survival curves for all-cause, CVD, and IHD mortality by depressive symptom categories and log-rank tests for difference in cumulative mortality across depressive symptom categories.

Previous studies have indicated that depressive symptoms are associated with alcohol consumption and cardiovascular health^31,32^; alcohol consumption and cardiovascular health are associated with all-cause and CVD deaths.^33,34^ Because alcohol consumption and cardiovascular health might be mediators for associations between depression and all-cause and cardiovascular death, we performed mediation analyses to examine whether alcohol use and the 8 cardiovascular health metrics mediate associations between depressive symptoms and all-cause, CVD, and IHD mortality. The proportion of mediation (p) was calculated using the formula p = (1 – β_1_ / β_2_) × 100. β_1_ and β_2_ represent β coefficients of depressive symptoms from models with and without mediators included, respectively. The bootstrapping method was used to calculate 95% CIs of the proportion of mediation.

We conducted 3 sensitivity analyses. The first examined the association between depressive symptoms and mortality from all-cause, CVD, and IHD after excluding participants who died within the first year of follow-up because of the possibility that death during the early stage of follow-up might be due to concurrent terminal illness or severe disease other than depression. The second sensitivity analysis examined the association between depression and mortality, including participants with history of CVD or cancer. The third sensitivity analysis examined the influences of imputing missing values of covariates on the association between depression and mortality. Multiple imputation (fully conditional specification) was used to impute missing data among participants who were eligible for the analysis (n = 27 566).^35^ Covariates used in the multiple imputation model included age, sex, race and Hispanic ethnicity, health insurance, marital status, education, poverty, family history of CVD, chronic kidney disease, depressive symptoms, antidepressant use, alcohol use, and the LE-8 metrics.

We used the first-day 24-hour dietary recall sampling weights and divided by 7 (data from 7 NHANES cycles) to represent the noninstitutionalized US population and account for sampling probability and nonresponse. Data were analyzed using SUDAAN version 11 (RTI International) to account for the complex survey design. All tests of statistical significance were 2-tailed, and a probability value <.05 was considered significant.

## Results

Of 34 648 nonpregnant adults aged 20 years and older with reliable first 24-hour dietary recall, we sequentially excluded 68 participants who were ineligible for mortality follow-up; 535 participants with BMI less than 18.5; 3953 participants with history of myocardial infarction, congestive heart failure, or stroke; and 2526 participants with history of cancer, yielding 27 566 participants eligible for analyses. We further excluded 1839 participants with missing depression scores, 1818 participants with missing LE-8 scores, and 215 participants with missing values for covariates. The final analysis included 23 694 adults.

About half of the participants were male (unweighted n = 11 862 [weighted 49.8%]), and the mean (SE) age was 44.7 (0.24) years. By unweighted self-report, 5004 participants were non-Hispanic Black (weighted 11.0%), 6545 were Hispanic (weighted 15.3%), 9607 were non-Hispanic White (weighted 66.2%), and 2538 were of another race or ethnicity (weighted 7.6%), including American Indian or Alaska Native, Asian, Native Hawaiian or Other Pacific Islander, or multiple races, consolidated owing to small numbers. The prevalence of none or minimal, mild, and moderate to severe depressive symptoms were 77.9%, 14.9%, and 7.2%, respectively. Participants who were female; never married or were divorced, widowed, or separated; had poverty index ratio less than 1.30; had less than 12 years of education, or had no health insurance were more likely to have self-reported depression. Adults with depression had significantly lower cardiovascular health scores in 6 of the LE-8 metrics (Table 1).

**Table 1.** Characteristics Across Depressive Symptoms in US Adults, National Health and Nutrition Examination Survey 2005-2018

Variable	All (N = 23 694)	Depressive symptoms, No. (%)			*P* value
		None (n = 18 344)	Mild (n = 3552)	Moderate to severe (n = 1798)	
Age, mean (SE), y	44.7 (0.24)	45.0 (0.25)	43.6 (0.44)	43.5 (0.50)	.006
Sex^a^					
Male	11 862 (49.8)	9655 (51.9)	1533 (44.1)	674 (38.9)	<.001
Female	11 832 (50.2)	8689 (48.1)	2019 (55.9)	1124 (61.1)	
Race and ethnicity^a^^,^^b^					
Non-Hispanic Black	5004 (11.0)	3851 (10.7)	765 (11.5)	388 (13.1)	
Hispanic	6545 (15.3)	5003 (14.8)	993 (16.2)	549 (18.1)	<.001
Non-Hispanic White	9607 (66.2)	7415 (66.9)	1458 (64.6)	734 (61.7)	
Other^c^	2538 (7.6)	2075 (7.6)	336 (7.7)	127 (7.0)	
Marital status^a^					
Never married	4740 (20.6)	3493 (19.6)	822 (23.5)	425 (24.9)	<.001
Married/living with partner	14 399 (63.0)	11 661 (65.9)	1912 (55.9)	826 (46.2)	
Divorced, separated, or widowed	4555 (16.4)	3190 (14.5)	818 (20.6)	547 (29.0)	
Poverty index ratio^a^^,^^d^					
<1.30	6356 (18.9)	4389 (16.2)	1177 (24.5)	790 (36.4)	<.001
1.30-3.49	8176 (32.4)	6341 (31.5)	1275 (36.3)	560 (33.3)	
≥3.5	7153 (42.2)	6092 (46.1)	796 (31.5)	265 (22.6)	
Missing	2009 (6.6)	1522 (6.3)	304 (7.7)	183 (7.7)	
Education level^a^					
<12 y	5450 (14.5)	3916 (13.1)	937 (17.9)	597 (22.3)	<.001
12 y	5427 (23.3)	4119 (22.5)	872 (26.0)	436 (26.5)	
>12 y	12 817 (62.2)	10 309 (64.4)	1743 (56.0)	765 (51.3)	
Alcohol use^a^					
None	13 144 (48.8)	10 051 (48.0)	1984 (49.2)	1109 (56.5)	.001
Moderate	8412 (40.3)	6775 (42.1)	1163 (36.6)	474 (29.5)	
Heavy	2138 (10.9)	1518 (10.0)	405 (14.3)	215 (14.0)	
Insurance status^a^					
Uninsured	5710 (19.3)	4181 (17.6)	983 (23.8)	546 (27.6)	<.001
Insured	17 984(80.7)	14 163 (82.4)	2569 (76.2)	1252 (72.4)	
Family history of CVD^a^					
Yes	2595 (11.8)	1766 (10.5)	495 (14.7)	334 (19.5)	<.001
No	21 099 (88.2)	16 578 (89.5)	3057 (85.3)	1464 (80.5)	
Chronic kidney disease^a^					
Yes	3219 (10.9)	2399 (10.7)	528 (11.9)	292 (11.4)	.25
No	20 475 (89.1)	15 945 (89.3)	3024 (88.1)	1506 (88.6)	
Antidepressant use^a^					
Yes	2173 (11.6)	1080 (8.0)	568 (20.3)	525 (33.4)	<.001
No	21 521 (88.4)	17 264 (92.0)	2984 (79.7)	1273 (66.6)	
LE-8 individual scores, mean (SE)					
Body mass index^e^	60.5 (0.46)	61.8 (0.51)	56.3 (0.91)	54.7 (1.22)	<.001
Nicotine exposure	71.7 (0.48)	75.0 (0.48)	63.8 (1.09)	52.5 (1.77)	<.001
Physical activity	53.0 (0.84)	56.0 (0.83)	46.3 (1.33)	34.2 (1.70)	<.001
Hypertension	70.7 (0.37)	70.9 (0.42)	70.1 (0.78)	69.8 (1.19)	.43
Diabetes	80.5 (0.27)	80.8 (0.33)	80.1 (0.61)	78.1 (0.83)	.004
Non-HDL cholesterol	64.4 (0.38)	64.7 (0.42)	63.0 (0.84)	63.7 (1.24)	.41
Diet	39.3 (0.51)	40.9 (0.54)	36.0 (0.84)	29.4 (1.03)	<.001
Sleep health	83.4 (0.28)	85.6 (0.25)	78.5 (0.70)	69.6 (1.26)	<.001
LE-8 summary score, mean (SE)	65.4 (0.30)	67.0 (0.30)	61.8 (0.46)	56.5 (0.56)	<.001
LE-8 levels^a^					
Low	4539 (16.2)	2968 (13.3)	928 (23.4)	643 (32.9)	<.001
Moderate	15 334 (64.1)	12 071 (64.9)	2227 (62.1)	1036 (59.4)	
High	3821 (19.7)	3305 (21.8)	397 (14.5)	119 (7.6)	

Abbreviations: CVD, cardiovascular disease; HDL, high-density lipoprotein; LE-8, Life’s Essential 8.

^a^ Unweighted numbers and weighted percentages shown.

^b^ Race and ethnicity were assessed as these variables are predictors of mortality and are associated with depression and other covariates.

^c^ Other race includes non-Hispanic Asian, non-Hispanic Native Hawaiian or Other Pacific Islander, non-Hispanic American Indian or Alaska Native, or multiracial, consolidated owing to small numbers.

^d^ Poverty-to-income ratio is the ratio of family income to the Department of Health and Human Services poverty measure.

There were 1495 total, 497 CVD, and 209 IHD deaths over 182 896 person-years of follow-up (mean [SE] follow-up, 7.72 [0.09] years). The weighted all-cause mortality rates per 1000 person-years were 5.62, 7.79, and 9.48, respectively, for none or minimal, mild, and moderate to severe depressive symptoms. The Figure shows the Kaplan-Meier survival curves for all-cause, CVD, and IHD mortality among adults with none or minimal, mild, and moderate to severe depressive symptoms. In fully adjusted models, depressive symptoms were independently associated with a significantly higher risk of all-cause mortality (Table 2). Compared to those without depressive symptoms, adults with mild depressive symptoms had a hazard ratio of 1.35 (95% CI, 1.07-1.72), and adults with moderate-to-severe depressive symptoms had a hazard ratio of 1.62 (95% CI, 1.24-2.12). Similar patterns were observed for CVD and IHD mortality except mild depression on IHD mortality (Table 2). The corresponding hazard ratios of CVD mortality were 1.49 (95% CI, 1.11-2.00) for mild depression and 1.79 (95% CI, 1.22-2.62) for moderate to severe depression. For IHD mortality, the hazard ratios were 0.96 (95% CI, 0.58-1.60) for mild depression and 2.21 (95% CI, 1.24-3.91) for moderate to severe depression, respectively. The associations were largely consistent by age, sex, diabetes status, and poverty subgroups (Table 3).

**Figure.**
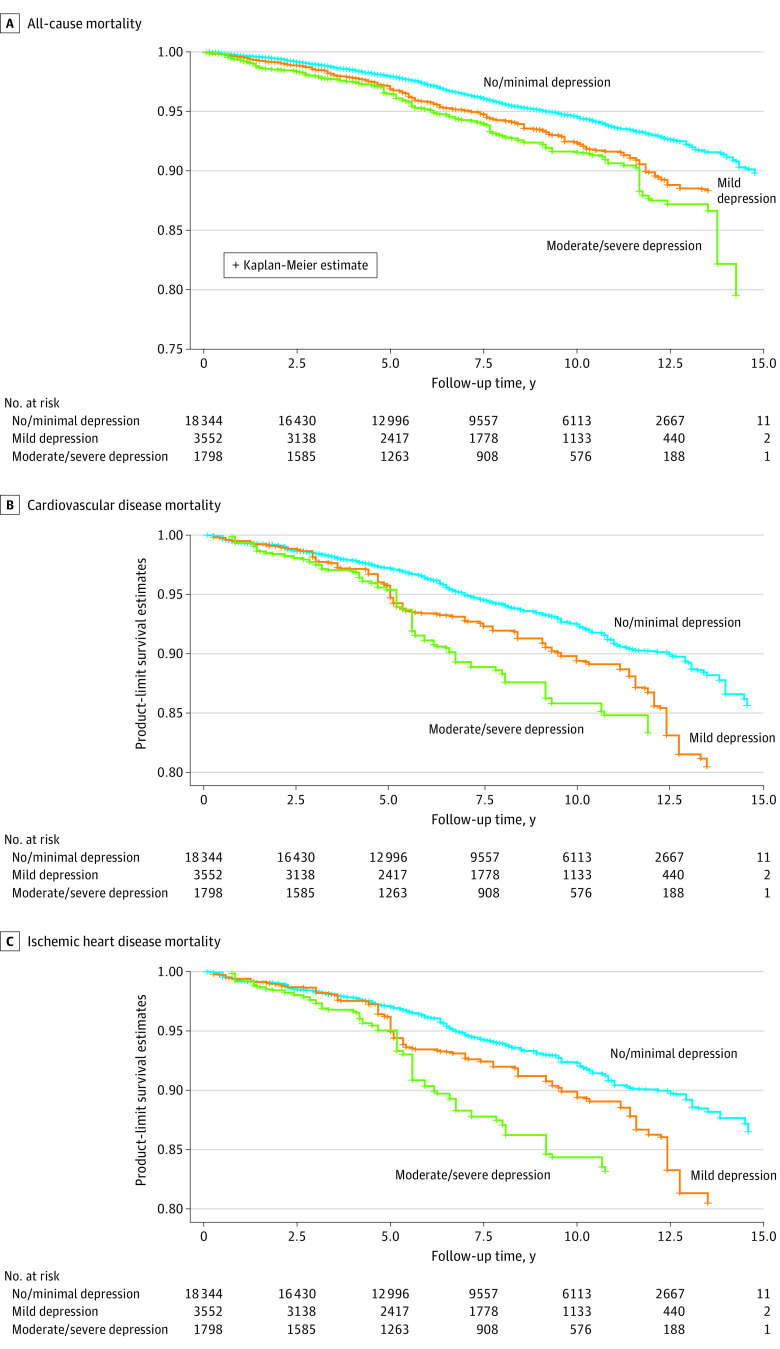
Kaplan-Meier Survival Curves by Depressive Symptoms National Health and Nutrition Examination Survey 2005-2018–Linked Mortality File Through 2019

**Table 2.** Depressive Symptoms for Death From All Cause, Cardiovascular Disease, and Ischemic Heart Disease in US Adults, National Health and Nutrition Examination Survey 2005-2018–Linked Mortality Files Through 2019

Variable	Depressive symptoms, hazard ratio (95% CI)		
	None	Mild	Moderate to severe
No. of participants	18 344	3552	1798
Person-y of follow-up	142 600	26 782	13 514
**All-cause mortality**			
No. of deaths	1100	251	144
Weighted mortality rate per 1000 person-y	5.62 (5.14-6.08)	7.79 (6.38-9.12)	9.48 (7.50-11.25)
Model 1^a^	1.00 [Reference]	1.72 (1.35-2.19)	2.50 (1.94-3.22)
Model 2^b^	1.00 [Reference]	1.42 (1.12-1.81)	1.78 (1.37-2.32)
Model 3^c^	1.00 [Reference]	1.35 (1.07-1.72)	1.62 (1.24-2.12)
**Cardiovascular disease mortality**			
No. of deaths	356	91	50
Weighted -mortality rate per 1000 person-y	1.64 (1.44-1.84)	2.42 (1.83-2.98)	2.72 (1.69-3.64)
Model 1^a^	1.00 [Reference]	1.90 (1.42-2.55)	2.78 (1.89-4.09)
Model 2^b^	1.00 [Reference]	1.57 (1.19-2.07)	1.94 (1.33-2.82)
Model 3^c^	1.00 [Reference]	1.49 (1.11-2.00)	1.79 (1.22-2.62)
**Ischemic heart disease mortality**			
No. of deaths	145	37	27
Weighted mortality rate per 1000 person-y	0.78 (0.62-0.93)	0.77 (0.49-1.04)	1.69 (0.73-2.55)
Model 1^a^	1.00 [Reference]	1.27 (0.78-2.08)	3.50 (1.93-6.35)
Model 2^b^	1.00 [Reference]	1.05 (0.65-1.71)	2.51 (1.43-4.40)
Model 3^c^	1.00 [Reference]	0.96 (0.58-1.60)	2.21 (1.24-3.91)

^a^ Model 1: adjusted for age, sex, race, and ethnicity.

^b^ Model 2: adjusted as in model 1 as well as for marital status, education, poverty, health insurance, chronic kidney disease, family history of cardiovascular disease, and antidepressant use.

^c^ Model 3: adjusted as in models 1 and 2 as well as for alcohol use and the 8 cardiovascular health metrics developed by the American Heart Association.

**Table 3.** Depressive Symptoms for Death From All Cause, Cardiovascular Disease, and Ischemic Heart Disease by Selected Characteristics in US Adults, National Health and Nutrition Examination Survey 2005-2018–Linked Mortality Files Through 2019^a^

Variable	Depressive symptoms, adjusted hazard ratio (95% CI)		
	None	Mild	Moderate to severe
**Age 20-64 y (n = 19 820)**			
All-cause mortality	1.00 [Reference]	1.35 (0.93-1.97)	1.73 (1.16-2.56)
Cardiovascular disease mortality	1.00 [Reference]	1.34 (0.67-2.68)	1.78 (0.90-3.58)
Ischemic heart disease mortality	1.00 [Reference]	0.58 (0.26-1.32)	1.67 (0.58-4.76)
**Age ≥65 y (n = 3874)**			
All-cause mortality	1.00 [Reference]	1.27 (0.99-1.62)	1.42 (1.00-2.02)
Cardiovascular disease mortality	1.00 [Reference]	1.47 (1.07-2.03)	1.77 (1.08-2.89)
Ischemic heart disease mortality	1.00 [Reference]	1.28 (0.73-2.25)	3.00 (1.58-5.69)
**Male (n = 11 862)**			
All-cause mortality	1.00 [Reference]	1.33 (0.95-1.88)	1.49 (0.98-2.27)
Cardiovascular disease mortality	1.00 [Reference]	1.70 (0.98-2.94)	1.32 (0.63-2.80)
Ischemic heart disease mortality	1.00 [Reference]	1.08 (0.49-2.38)	1.81 (0.70-4.73)
**Female (n = 11 832)**			
All-cause mortality	1.00 [Reference]	1.37 (1.02-1.83)	1.73 (1.19-2.50)
Cardiovascular disease mortality	1.00 [Reference]	1.33 (0.91-1.95)	2.07 (1.34-3.20)
Ischemic heart disease mortality	1.00 [Reference]	0.79 (0.41-1.59)	2.52 (1.20-5.29)
**Diabetes: yes (n = 3357)**			
All-cause mortality	1.00 [Reference]	1.70 (1.20-2.42)	1.31 (0.81-2.11)
Cardiovascular disease mortality	1.00 [Reference]	1.76 (1.05-2.93)	1.53 (0.79-2.96)
Ischemic heart disease mortality	1.00 [Reference]	0.99 (0.43-2.30)	1.40 (0.52-3.78)
**Diabetes: no (n = 20 337)**			
All-cause mortality	1.00 [Reference]	1.25 (0.95-1.63)	1.75 (1.26-2.44)
Cardiovascular disease mortality	1.00 [Reference]	1.46 (1.01-2.11)	1.97 (1.22-3.18)
Ischemic heart disease mortality	1.00 [Reference]	1.00 (0.57-1.74)	2.70 (1.40-5.21)
**Poverty index ratio <1.30 (n = 6356)** ^b^			
All-cause mortality	1.00 [Reference]	1.34 (0.98-1.83)	1.41 (0.98-2.02)
Cardiovascular disease mortality	1.00 [Reference]	1.44 (0.87-2.41)	1.65 (0.94-2.92)
Ischemic heart disease mortality	1.00 [Reference]	0.82 (0.40-1.66)	1.83 (0.72-4.68)
**Poverty index ratio 1.30-3.49 (n = 8176)** ^b^			
All-cause mortality	1.00 [Reference]	1.30 (0.96-2.78)	1.34 (0.83-2.16)
Cardiovascular disease mortality	1.00 [Reference]	1.84 (1.16-2.91)	1.46 (0.60-3.58)
Ischemic heart disease mortality	1.00 [Reference]	1.40 (0.71-2.74)	2.79 (1.06-7.35)
**Poverty index ratio ≥3.50 (n = 7153)** ^b^			
All-cause mortality	1.00 [Reference]	1.39 (0.82-2.36)	2.00 (0.89-4.51)
Cardiovascular disease mortality	1.00 [Reference]	0.70 (0.27-1.83)	2.03 (0.70-5.90)
Ischemic heart disease mortality	1.00 [Reference]	0.41 (0.10-1.79)	1.98 (0.53-7.40)

^a^ Adjusted for age, sex, race, ethnicity, marital status, education, poverty, chronic kidney disease, family history of cardiovascular disease, antidepressant use, alcohol use, and the 8 cardiovascular health metrics developed by the American Heart Association.

^b^ Poverty-to-income ratio is the ratio of family income to the Department of Health and Human Services poverty measure.

Feeling tired or having little energy was the most common individual depressive symptom, experienced by 50.1% (weighted) of the sample (unweighted n = 11 352 of 23 694). Other common depressive symptoms included trouble sleeping or sleeping too much (unweighted n = 8592 [weighted 38.5%]); poor appetite or overeating (unweighted n = 5523 [weighted 23.0%]); having little interest in doing things (unweighted n = 5528 [weighted 22.6%]); and feeling down, depressed, or hopeless (unweighted n = 5441 [weighted 22.2%]) (eTable 2 in Supplement 1). At the highest severity (nearly every day during the past 2 weeks), feeling tired or having little energy, poor appetite or overeating, and having little interest in doing things were significantly associated with all-cause and CVD mortality after adjusting for potential confounders (eTable 2 in Supplement 1).

Mediators considered in this analysis explained 14.2% and 11.0% of the association between mild depressive symptoms and all-cause and CVD mortality, and 16.1%, 12.0%, and 14.3% between moderate to severe depressive symptoms and all-cause, CVD, and IHD mortality, respectively (Table 4). The important mediators of all-cause mortality included smoking, physical activity, and sleep health. Physical activity, smoking, diabetes, and BMI were important mediators of CVD and IHD mortality.

**Table 4.** Mediation of Alcohol Use and 8 Cardiovascular Health Metrics on Association Between Depressive Symptoms and Death From All-Cause, Cardiovascular Disease, and Ischemic Heart Disease in US Adults, National Health and Nutrition Examination Survey 2005-2018–Linked Mortality Files Through 2019

Variable	Depressive symptoms, hazard ratio (95% CI)			Proportion of mediating effect (95% CI)	
	None	Mild	Moderate to severe	Mild vs no depressive symptoms	Moderate to severe vs no depressive symptoms
**All-cause mortality**					
Base model^a^	1.00 [Reference]	1.42 (1.12 to 1.81)	1.78 (1.37 to 2.32)	NA	NA
Base model + alcohol use	1.00 [Reference]	1.42 (1.12 to 1.82)	1.78 (1.37 to 2.32)	3.27 (2.61 to 6.19)	2.88 (−1.82 to 3.62)
Base model + smoking	1.00 [Reference]	1.36 (1.07 to 1.73)	1.65 (1.27 to 2.14)	13.67 (12.62 to 14.28)	12.96 (12.48 to 13.46)
Base model + physical activity	1.00 [Reference]	1.37 (1.07 to 1.75)	1.67 (1.28 to 2.18)	10.95 (10.34 to 11.50)	10.64 (10.25 to 11.05)
Base model + diet	1.00 [Reference]	1.42 (1.11 to 1.80)	1.75 (1.35 to 2.29)	1.77 (1.50 to 2.03)	2.56 (2.29 to 2.85)
Base model + body mass index^b^	1.00 [Reference]	1.43 (1.12 to 1.82)	1.78 (1.37 to 2.33)	0.36 (0.21 to 0.43)	0.40 (0.27 to 0.52)
Base model + hypertension	1.00 [Reference]	1.42 (1.12 to 1.81)	1.80 (1.38 to 2.34)	0.32 (0.01 to 0.49)	−1.63 (−1.97 to −1.27)
Base model + non-HDL cholesterol	1.00 [Reference]	1.43 (1.13 to 1.82)	1.78 (1.37 to 2.31)	1.54 (1.75 to 1.35)	0.11 (−0.10 to 0.36)
Base model + diabetes	1.00 [Reference]	1.41 (1.11 to 1.79)	1.73 (1.33 to 2.26)	3.11 (2.77 to 3.56)	4.41 (4.11 to 5.00)
Base model + sleep health	1.00 [Reference]	1.38 (1.08 to 1.77)	1.67 (1.28 to 2.17)	8.32 (7.80 to 8.93)	11.24 (10.27 to 11.12)
Base model + alcohol use and 8 cardiovascular health metrics	1.00 [Reference]	1.37 (1.08 to 1.74)	1.65 (1.26 to 2.17)	14.17 (13.16 to 14.87)	16.08 (15.40 to 16.98)
**Cardiovascular disease mortality**					
Base model^a^	1.00 [Reference]	1.57 (1.19 to 2.07)	1.94 (1.33 to 2.82)		
Base model + alcohol use	1.00 [Reference]	1.57 (1.19 to 2.07)	1.94 (1.33 to 2.82)	1.66 (1.37 to 2.10)	1.51 (1.32 to 1.84)
Base model + smoking	1.00 [Reference]	1.54 (1.16 to 2.03)	1.89 (1.31 to 2.73)	4.27 (4.10 to 4.53)	3.67 (3.39 to 3.96)
Base model + physical activity	1.00 [Reference]	1.51 (1.13 to 2.01)	1.84 (1.25 to 2.70)	8.22 (7.85 to 8.94)	7.69 (6.94 to 8.55)
Base model + diet	1.00 [Reference]	1.56 (1.18 to 2.05)	1.91 (1.32 to 2.79)	1.70 (1.18 to 2.43)	1.70 (1.23 to 2.49)
Base model + body mass index^b^	1.00 [Reference]	1.55 (1.17 to 2.06)	1.90 (1.29 to 2.81)	1.96 (1.75 to 2.18)	2.59 (2.18 to 2.75)
Base model + hypertension	1.00 [Reference]	1.56 (1.18 to 2.08)	1.98 (1.34 to 2.92)	0.68 (0.24 to 1.03)	−3.60 (−4.40 to −3.18)
Base model + non-HDL cholesterol	1.00 [Reference]	1.56 (1.18 to 2.07)	1.94 (1.33 to 2.88)	0.53 (0.40 to 0.64)	−0.01 (−0.10 to 0.07)
Base model + diabetes	1.00 [Reference]	1.55 (1.18 to 2.05)	1.89 (1.29 to 2.77)	1.95 (1.72 to 2.23)	3.48 (2.99 to 3.87)
Base model + sleep health	1.00 [Reference]	1.56 (1.18 to 2.05)	1.91 (1.33 to 2.74)	1.27 (1.05 to 1.55)	1.89 (1.50 to 2.34)
Base model + alcohol use and 8 cardiovascular health metrics	1.00 [Reference]	1.50 (1.12 to 2.01)	1.80 (1.22 to 2.66)	10.97 (10.20 to 11.78)	11.98 (11.23 to 12.56)
**Ischemic heart disease mortality**					
Base model^a^	1.00 [Reference]	1.05 (0.65 to 1.71)	2.51 (1.43 to 4.40)		
Base model + alcohol use	1.00 [Reference]	1.05 (0.64 to 1.71)	2.51 (1.44 to 4.38)	NA	2.27 (1.86 to 2.68)
Base model + smoking	1.00 [Reference]	1.00 (0.62 to 1.63)	2.38 (1.36 to 4.15)	NA	5.94 (5.61 to 6.55)
Base model + physical activity	1.00 [Reference]	1.00 (0.61 to 1.63)	2.35 (1.33 to 4.15)	NA	7.50 (6.88 to 8.42)
Base model + diet	1.00 [Reference]	1.04 (0.65 to 1.69)	2.48 (1.42 to 4.31)	NA	1.58 (1.27 to 1.95)
Base model + body mass index^b^	1.00 [Reference]	1.05 (0.64 to 1.71)	2.48 (1.38 to 4.46)	NA	1.59 (1.30 to 1.86)
Base model + hypertension	1.00 [Reference]	1.05 (0.64 to 1.72)	2.54 (1.44 to 4.50)	NA	−1.44 (−1.81 to −1.21)
Base model + non-HDL cholesterol	1.00 [Reference]	1.04 (0.63 to 1.71)	2.51 (1.42 to 4.42)	NA	0.29 (−0.02 to 0.48)
Base model + diabetes	1.00 [Reference]	1.04 (0.64 to 1.69)	2.47 (1.40 to 4.34)	NA	1.94 (1.60 to 2.33)
Base model + sleep health	1.00 [Reference]	1.06 (0.66 to 1.71)	2.55 (1.52 to 4.29)	NA	−1.85 (−2.98 to 0.43)
Base model + alcohol use and 8 cardiovascular health metrics	1.00 [Reference]	0.95 (0.59 to 1.62)	2.25 (1.25 to 4.04)	NA	14.27 (12.96 to 15.24)

Abbreviations: HDL, high-density lipoprotein; NA, not applicable.

^a^ Adjusted for age, sex, race, ethnicity, marital status, education, poverty, chronic kidney disease, family history of cardiovascular disease, and antidepressant use.

^b^ Calculated as weight in kilograms divided by height in meters squared.

Associations between depressive symptoms and mortality remained largely consistent after excluding participants who died during the first year of follow-up (eTable 3 in Supplement 1) or when including participants with history of CVD or cancer (eTable 4 in Supplement 1). The baseline characteristics by depressive symptoms after multiple imputation (eTable 5 in Supplement 1) were comparable to those generated from participants with complete data (Table 1), and associations between depression and mortality were similar (eTable 6 in Supplement 1).

## Discussion

In this nationally representative cohort study, we found that 7.2% of US adults had moderate to severe depressive symptoms, and 14.9% had mild depressive symptoms. Compared to those without depressive symptoms, participants with mild depressive symptoms had a 35% and 49% higher risk of all-cause and CVD mortality, respectively. Risk of all-cause, CVD, and IHD mortality was 62%, 79%, and 121% higher, respectively, for those with moderate to severe depressive symptoms compared to those without depressive symptoms. Associations were largely consistent across subgroups and in all sensitivity analyses.

Our findings about overall associations between depression and mortality risk are consistent with previous study results.^5,6,7,8,9,10,12,13,14,15,16,17,18,19,20^ A study using the 1999 National Health Interview Survey linked with mortality data through 2011 showed that, adjusting for demographic factors, anxiety or depression was associated with 61% elevated risk of mortality.^10^ A cohort study in Canada reported that the association between depression and mortality persisted over 3 time periods from 1952 to 1992.^8^ An analysis from Central and Eastern Europe indicated that depressive symptoms estimated CVD and all-cause mortality independently of various potential confounders.^9^ A recent study using 2 large cohorts in China reported similar association between depression and mortality.^15^

In contrast to our study, previous studies have reported that associations of depression and mortality differed by gender. A study using NHANES I data indicated that depression was associated with an increased risk of IHD mortality in men, but not in women.^4^ A recent study in China also showed the depression-mortality association was more evident in men.^15^ While the present study did not find statistically significant interaction by sex, women had a higher prevalence of depression, and hazard ratios for associations between depression and all-cause, CVD, and IHD mortality were higher in women compared to men. It is possible that there was differential misclassification of self-reported depression by sex, especially if men were more hesitant to report depressive symptoms than women.^36^

Prior studies have also observed differential associations of depression and mortality by diabetes status and income levels, which were not replicated in our study. For example, 1 study^25^ showed that diabetes modified the depression-mortality association, with an association between depression and mortality only observed among persons with diabetes. Another study^37^ found that elevated depressive symptoms and perceived stress were associated with increased risk of first-onset CVD events, including cardiovascular death, in participants with low income but not in those with high income. Our analyses of the association between depressive symptoms and mortality did not find subgroup differences. The discrepancies between previous studies and the current study might be due to differences in instruments assessing depressive symptoms, classification of depressive symptom severity, or confounders included in the models.

Several mechanisms have been suggested for the association between depressive symptoms and all-cause and CVD mortality.^38^ Depression may affect lifestyle factors, such as obesity, physical inactivity, smoking, unhealthy diet, and excessive alcohol use, which are associated with an increase in risk of cardiovascular events and all-cause deaths.^39,40,41,42^ Compared to those without depressive symptoms, participants with depressive symptoms had increased CVD risk profiles in this study population, and our results estimated that 11% to 16% of the associations between depressive symptoms and all-cause and CVD deaths could be explained by alcohol use and 8 cardiovascular health metrics. Social factors such as poverty, housing instability, lower educational attainment, lower income, and lack of health insurance are related to depression and may play an important role in the depression-mortality association.^43^ In addition, several biological mechanisms have been proposed, such as chronic inflammation, hypothalamic-pituitary-adrenal (HPA)-axis dysregulation, autonomic cardiac dysregulation after antidepressant use, neurotransmitters, neurocircuitry, clinical metabolic dysregulation, and coronary artery endothelial dysfunction.^44,45,46^ For example, neuroendocrinologically, anhedonia (loss of interest in doing things) has been related to dysfunctional feedback between the HPA axis and specific brain regions.^47,48^ Increased HPA axis activity could lead to higher levels of circulating cortisol, which could affect blood pressure and vascular endothelial function through the vascular nitric oxide system.^49^ In addition, anhedonia is associated with catecholaminergic dysfunction.^50^ High levels of catecholamine can trigger tachyarrhythmia^51,52^ and promote platelet aggregation.^53,54^

### Limitations

Some limitations must be considered when interpreting our results. First, depressive symptoms were measured only at baseline; therefore, our analyses were not able to account for the changes in depressive symptoms over time. Second, persons with severe depression may have disproportionately chosen not to participate in the survey or health examination; therefore, our prevalence estimates may underestimate the actual prevalence of depression. In addition, participants being successfully treated for depression would not be identified as having depression by the PHQ-9. Third, PHQ-9–based depressive symptoms, rather than clinical depression, can be highly variable. There could be differences in interpreting and answering the questions, especially for participants with different backgrounds and beliefs. However, PHQ-9 scores of 10 or higher have both sensitivity and specificity of 88% for major depression.^31^ Fourth, we cannot fully exclude potential effects of unmeasured confounding factors—such as anxiety disorder, general stress, and work-related stress—so the association between depression and mortality could be overestimated in our analysis. Fifth, among 27 566 participants who were eligible for the mortality analyses, 14% had missing data on depression and other covariates, which could possibly overestimate or underestimate the association between depression and mortality. However, multiple imputation methods showed consistent results.

## Conclusions

There was a graded positive association between mild and moderate to severe depressive symptoms and mortality in this study. Our analyses extend the findings from previous studies by assessing these associations in a large, diverse, and nationally representative sample and by using more nuanced CVD-related causes of death. Taken together with the body of literature on associations between depression and CVD mortality, these findings can support public health efforts to develop a comprehensive, nationwide strategy to improve well-being, including both mental and cardiovascular health.

## Notes

Supplement 1. **eTable 1.** Definition and scoring approach for quantifying the American Heart Association’s Life’s Essential 8 cardiovascular health score, NHANES 2005-2018 **eTable 2.** Adjusted hazard ratios and 95% CI of 9 depressive symptoms for death from all-cause, cardiovascular disease, and ischemic heart disease in US adults, NHANES 2005-2018 Linked Mortality File Through 2019 **eTable 3.** Hazard ratios and 95% CI of depressive symptoms for death from all-cause, cardiovascular disease, and ischemic heart disease—excluding participants who died within 1 year of follow-up in US adults, NHANES 2005-2018 Linked Mortality File Through 2019 **eTable 4.** Hazard ratios and 95% CI of depressive symptoms for death from all-cause, cardiovascular disease, and ischemic heart disease—including participants with history of cardiovascular disease and cancer, in US adults, NHANES 2005-2018 Linked Mortality File Through 2019 **eTable 5.** Characteristics across depressive symptoms after multiple imputation among participants eligible for mortality analyses (n=27 566), NHANES 2005-2018 **eTable 6.** Unadjusted and adjusted hazard ratios and 95% CI of depressive symptoms for death from all cause, cardiovascular disease and ischemic heart disease after multiple imputation among participants eligible for mortality analyses (n=27 566), NHANES 2005-2018 Linked Mortality File Through 2019

Supplement 2.
Data Sharing Statement
