# Supplementary material for: Depressive Symptoms and Mortality Among US Adults
Source: JAMA Netw Open. 2023 Oct 9;6(10):e2337011. doi: 10.1001/jamanetworkopen.2023.37011 (PMC10562940; doi:10.1001/jamanetworkopen.2023.37011)
Supplement: Supplement 1. — eTable 1. Definition and scoring approach for quantifying the American Heart Association’s Life’s Essential 8 cardiovascular health score, NHANES 2005-2018 eTable 2. Adjusted hazard ratios and 95% CI of 9 depressive symptoms for death from all-cause, cardiovascular disease, and ischemic heart disease in US adults, NHANES 2005-2018 Linked Mortality File Through 2019 eTable 3. Hazard ratios and 95% CI of depressive symptoms for death from all-cause, cardiovascular disease, and ischemic heart disease—excluding participants who died within 1 year of follow-up in US adults, NHANES 2005-2018 Linked Mortality File Through 2019 eTable 4. Hazard ratios and 95% CI of depressive symptoms for death from all-cause, cardiovascular disease, and ischemic heart disease—including participants with history of cardiovascular disease and cancer, in US adults, NHANES 2005-2018 Linked Mortality File Through 2019 eTable 5. Characteristics across depressive symptoms after multiple imputation among participants eligible for mortality analyses (n=27 566), NHANES 2005-2018 eTable 6. Unadjusted and adjusted hazard ratios and 95% CI of depressive symptoms for death from all cause, cardiovascular disease and ischemic heart disease after multiple imputation among participants eligible for mortality analyses (n=27 566), NHANES 2005-2018 Linked Mortality File Through 2019 [file jamanetwopen-e2337011-s001.pdf]

Supplemental Online Content

Zhang Z, Jackson S, Gillespie C, Merritt R, Yang Q. Depressive symptoms and mortality among US adults. *JAMA Netw Open*. 2023;6(10):e2337011 doi:10.1001/jamanetworkopen.2023.37011

eTable 1. Definition and scoring approach for quantifying the American Heart Association's Life's Essential 8 cardiovascular health score, NHANES 2005-2018

eTable 2. Adjusted hazard ratios and 95% CI of 9 depressive symptoms for death from all-cause, cardiovascular disease, and ischemic heart disease in US adults, NHANES 2005-2018 Linked Mortality File Through 2019

eTable 3. Hazard ratios and 95% CI of depressive symptoms for death from all-cause, cardiovascular disease, and ischemic heart disease—excluding participants who died within 1 year of follow-up in US adults, NHANES 2005-2018 Linked Mortality File Through 2019

eTable 4. Hazard ratios and 95% CI of depressive symptoms for death from all-cause, cardiovascular disease, and ischemic heart disease—including participants with history of cardiovascular disease and cancer, in US adults, NHANES 2005-2018 Linked Mortality File Through 2019

eTable 5. Characteristics across depressive symptoms after multiple imputation among participants eligible for mortality analyses (n=27 566), NHANES 2005-2018

eTable 6. Unadjusted and adjusted hazard ratios and 95% CI of depressive symptoms for death from all cause, cardiovascular disease and ischemic heart disease after multiple imputation among participants eligible for mortality analyses (n=27 566), NHANES 2005-2018 Linked Mortality File Through 2019

This supplemental material has been provided by the authors to give readers additional information about their work.

© 2023 Zhang Z et al. *JAMA Network Open.*

eTable 1. Definition and scoring approach for quantifying the American Heart Association's Life's Essential 8 cardiovascular health score, NHANES 2005-2018

CVH metric	Method of measurement	Quantification of CVH metric, adults (≥20 years)	Quantification of CVH metric, children (up to 19 years)
**Diet**	**Measurement**: Self-reported daily intake of a DASH-style eating pattern<br>**Example tools for measurement:** Healthy Eating Index-2015 score[a]	Quantiles of Healthy Eating Index-2015 score<br>**Scoring:**<br>Points Quantile<br>100: ≥95th %ile (top/ideal diet)<br>80: 75th - 94th %ile<br>50: 50th - 74th %ile<br>25: 25th - 49th %ile<br>0: 1st - 24th %ile (bottom/least ideal quartile)	Quantiles of Healthy Eating Index-2015 score; ages 2-19<br>**Scoring:**<br>Points Quantile<br>100: ≥95th %ile (top/ideal diet)<br>80: 75th - 94th %ile<br>50: 50th - 74th %ile<br>25: 25th - 49th %ile<br>0: 1st - 24th %ile (bottom/least ideal quartile)
**Physical activity**	**Measurement:** Self-reported minutes of moderate or vigorous physical activity per week<br>**Example tools for measurement:** NHANES PAQ questionnaire	**Metric:** Minutes of moderate (or greater) intensity activity per week<br>**Scoring:**<br>Points Minutes<br>100: ≥150<br>90: 120-149<br>80: 90-119<br>60: 60-89<br>40: 30-59<br>20: 1-29<br>0: 0	**Metric:** Minutes of moderate (or greater) intensity activity per week; ages 2-19 years<br>**Scoring:**<br>Points Minutes<br>100: ≥420<br>90: 360-419<br>80: 300-359<br>60: 240-299<br>40: 120-239<br>20: 1-119<br>0: 0
**Nicotine exposure**	**Measurement:** Self-reported use of cigarettes or inhaled nicotine-delivery system<br>**Example tools for measurement:** NHANES SMQ	**Metric:** Combustible tobacco use and/or inhaled nicotine-delivery system use; or secondhand smoke exposure<br>**Scoring:**<br>Points Status<br>100: Never smoker<br>75: Former smoker, quit ≥5 y<br>50: Former smoker, quit 1 - <5 y<br>25: Former smoker, quit <1 y, or currently using inhaled nicotine-delivery system	**Metric:** Combustible tobacco use and/or inhaled nicotine-delivery system use, or secondhand smoke exposure; ages 12-19<br>**Scoring:**<br>Points Status<br>100: Never tried<br>50: Tried any nicotine product, but >30 d ago<br>25: Currently using inhaled nicotine-delivery system

© 2023 Zhang Z et al. *JAMA Network Open.*

		0: Current smoker<br>Subtract 20 points (unless score is 0) for living with active indoor smoker in home	0: Current combustible use (any within 30 days)<br>Subtract 20 points (unless score is 0) for living with active indoor smoker in home
**Sleep health**	**Measurement:** Self-reported average hours of sleep per night<br>**Example tools for measurement:** "On average, how many hours of sleep do you get per night?" Consider objective sleep/actigraphy data from wearable technology, if available	**Metric:** Average hours of sleep per night<br>**Scoring:**<br>Points Level<br>100: 7-<9<br>90: 9-<10<br>70: 6-<7<br>40: 5-<6 or ≥10<br>20: 4-<5<br>0: <4	**Metric:** Average hours of sleep per night; ages 16-19<br>**Scoring:**<br>Points Level<br>100: Age-appropriate optimal range<br>90: <1 h above optimal range<br>70: <1 h below optimal range<br>40: 1-<2 h below or ≥1 h above optimal<br>20: 2-<3 h below optimal range<br>0: ≥3 h below optimal range
**Body mass index**	**Measurement:** Body weight (kg) divided by height squared (m^2)<br>**Example tools for measurement:** Objective measurement of height and weight	**Metric:** Body mass index (kg/m^2)<br>**Scoring:**<br>Points Level<br>100: <25<br>70: 25.0-29.9<br>30: 30.0-34.9<br>15: 35.0-39.9<br>0: ≥40.0	**Metric:** Body mass index percentiles (%iles) for age and sex; ages 2-19<br>**Scoring:**<br>Points Level<br>100: 5th-<85th %ile<br>70: 85th-<95th %ile<br>30: 95th %ile-<120% of the 95th %ile<br>15: 120% of the 95th %ile-<140% of the 95th %ile<br>0: ≥140% of the 95th %ile
**Blood lipids**	**Measurement:** Plasma total and HDL-cholesterol with calculation of non-HDL-cholesterol<br>**Example tools for measurement:** Fasting or non-fasting blood sample	**Metric:** Non-HDL-cholesterol (mg/dL)<br>**Scoring:**<br>Points Level<br>100: <130<br>60: 130-159<br>40: 160-189<br>20: 190-219<br>0: ≥220<br>If drug-treated level, subtract 20 points	**Metric:** Non-HDL cholesterol (mg/dL); ages 6-19<br>**Scoring:**<br>Points Level<br>100: <100<br>60: 100-119<br>40: 120-144<br>20: 145-189<br>0: ≥190<br>If drug-treated level, subtract 20 points
**Blood glucose**	**Measurement:** Fasting blood glucose[b] or casual hemoglobin A1c	**Metric:** Fasting blood glucose (mg/dL) or hemoglobin A1c (%)	**Metric:** Fasting blood glucose (mg/dL) or hemoglobin A1c (%); ages 12-19

© 2023 Zhang Z et al. *JAMA Network Open.*

	**Example tools for measurement:** Fasting blood glucose or hemoglobin A1c blood sample	**Scoring:**<br>Points Level<br>100: No history of diabetes and FBG <100 (or hemoglobin A1c <5.7)<br>60: No diabetes and FBG 100-125 (or hemoglobin A1c 5.7-6.4) (pre-diabetes)<br>40: Diabetes with hemoglobin A1c <7.0<br>30: Diabetes with hemoglobin A1c 7.0-7.9<br>20: Diabetes with hemoglobin A1c 8.0-8.9<br>10: Diabetes with hemoglobin A1c 9.0-9.9<br>0: Diabetes with hemoglobin A1c ≥10.0	**Scoring:**<br>Points Level<br>100: No history of diabetes and fasting FBG <100 (or hemoglobin A1c <5.7)<br>60: No diabetes and FBG 100-125 (or hemoglobin A1c 5.7-6.4) (pre-diabetes)<br>40: Diabetes with hemoglobin A1c <7.0<br>30: Diabetes with hemoglobin A1c 7.0-7.9<br>20: Diabetes with hemoglobin A1c 8.0-8.9<br>10: Diabetes with hemoglobin A1c 9.0-9.9<br>0: Diabetes with hemoglobin A1c ≥10.0
**Blood pressure**	**Measurement:** Appropriately measured systolic and diastolic blood pressure<br>**Example tools for measurement:** Appropriately sized blood pressure cuff	**Metric:** Systolic and diastolic blood pressure (mm Hg)<br>**Scoring:**<br>Points Status<br>100: <120/<80 (optimal)<br>75: 120-129/<80 (elevated)<br>50: 130-139 or 80-89 (Stage I HTN)<br>25: 140-159 or 90-99<br>0: ≥160 or ≥100<br>Subtract 20 points if treated level	**Metric:** Systolic and diastolic blood pressure (mm Hg) percentiles for ages 8-12. For ages ≥13, use adult scoring.<br>**Scoring:**<br>Points Status<br>100: Optimal (<90th %ile)<br>75: Elevated (≥90th-<95th %ile or ≥120/80 mm Hg to <95th %ile, whichever is lower)<br>50: Stage I HTN (≥95th-<95th %ile + 12 mmHg, or 130/80 - 139/89 mm Hg, whichever is lower)<br>25: Stage 2 HTN (≥95th %ile + 12 mm Hg, or<br>≥140/90 mm Hg, whichever is lower)<br>0: SBP ≥160 or ≥95th %ile + 30 mm Hg systolic, whichever is lower; and/or DBP ≥100 or ≥95th %ile + 20 mm Hg diastolic<br>Subtract 20 points if treated level

Abbreviations: %ile, percentile; CVH, cardiovascular health; DASH, Dietary Approaches to Stop Hypertension; DBP, diastolic blood pressure; FBG, fasting blood glucose; HDL, high density lipoprotein; HTN, hypertension; NHANES, National Health and Nutrition Examination Survey; PAQ, physical activity questionnaire; SBP, systolic blood pressure; SMQ, smoking and tobacco use questionnaire.

[a] The USDA Healthy Eating Index-2015 (HEI-2015) scores were based on 13 components: total fruit, whole fruit, total vegetables, grains and beans, whole grains, dairy, total protein foods, seafood and plant proteins, fatty acid, refined grains, sodium, added sugar, and saturated fats, with total scores ranging 0-100, and with a higher score indicating a healthier diet.

© 2023 Zhang Z et al. *JAMA Network Open.*

[b] The forward calibration equation was used to adjust data from the NHANES 2005-2014 cycles to be consistent with 2015-2018 cycles, to account for changes in glucose measurement methods over time.

© 2023 Zhang Z et al. *JAMA Network Open.*

eTable 2. Adjusted hazard ratios and 95% CI of 9 depressive symptoms for death from all-cause, cardiovascular disease, and ischemic heart disease in US adults, NHANES 2005-2018 Linked Mortality File Through 2019[a]

	Number of any symptoms (%)	Not at all	Several days/more than half the days	Nearly every day
**Have little interest in doing things**	5528 (22.6)			
All-cause mortality		1.00 (reference)	1.36 (1.09-1.69)	1.60 (1.14-2.23)
Cardiovascular disease mortality		1.00 (reference)	1.42 (1.06-1.90)	2.05 (1.25-3.34)
Ischemic heart disease mortality		1.00 (reference)	0.92 (0.57-1.47)	2.16 (0.97-4.82)
**Feeling down, depressed, or hopeless**	5441 (22.2)			
All-cause mortality		1.00 (reference)	1.23 (1.00-1.55)	1.08 (0.78-1.53)
Cardiovascular disease mortality		1.00 (reference)	1.32 (0.97-1.81)	1.21 (0.65-2.25)
Ischemic heart disease mortality		1.00 (reference)	1.05 (0.62-1.78)	1.27 (0.53-3.02)
**Trouble sleeping or sleeping too much**	8592 (38.5)			
All-cause mortality		1.00 (reference)	1.07 (0.92-1.26)	1.19 (0.92-1.54)
Cardiovascular disease mortality		1.00 (reference)	1.13 (0.84-1.52)	1.20 (0.80-1.80)
Ischemic heart disease mortality		1.00 (reference)	1.44 (0.89-2.33)	1.32 (0.70-2.50)
**Feeling tired or having little energy**	11 352 (50.1)			
All-cause mortality		1.00 (reference)	1.12 (0.95-1.31)	1.68 (1.24-2.27)
Cardiovascular disease mortality		1.00 (reference)	1.19 (0.93-1.52)	1.64 (1.16-2.33)
Ischemic heart disease mortality		1.00 (reference)	1.17 (0.75-1.81)	1.70 (0.95-3.05)
**Poor appetite or overeating**	5523 (23.0)			
All-cause mortality		1.00 (reference)	1.30 (1.03-1.65)	1.88 (1.36-2.60)
Cardiovascular disease mortality		1.00 (reference)	1.26 (0.82-1.93)	1.83 (1.19-2.81)
Ischemic heart disease mortality		1.00 (reference)	1.20 (0.54-2.66)	1.17 (0.49-2.80)
**Feeling bad about yourself**	3909 (16.9)			
All-cause mortality		1.00 (reference)	1.11 (0.87-1.40)	1.25 (0.80-1.94)
Cardiovascular disease mortality		1.00 (reference)	1.14 (0.83-1.56)	1.10 (0.54-2.24)
Ischemic heart disease mortality		1.00 (reference)	0.91 (0.52-1.60)	1.27 (0.58-2.79)
**Trouble concentrating on things**	3754 (16.8)			
All-cause mortality		1.00 (reference)	1.10 (0.87-1.40)	1.22 (0.85-1.74)
Cardiovascular disease mortality		1.00 (reference)	1.19 (0.78-1.83)	0.84 (0.40-1.79)
Ischemic heart disease mortality		1.00 (reference)	1.08 (0.61-1.91)	1.50 (0.67-3.39)
**Moving or speaking slowly or too fast**	2319 (9.0)			
All-cause mortality		1.00 (reference)	1.14 (0.87-1.48)	1.43 (0.90-2.27)

© 2023 Zhang Z et al. *JAMA Network Open.*

Cardiovascular disease mortality		1.00 (reference)	1.14 (0.77-1.69)	1.03 (0.49-2.13)
Ischemic heart disease mortality		1.00 (reference)	1.31 (0.70-2.47)	1.01 (0.29-3.46)
**Thought you would be better off dead**	773 (3.0)			
All-cause mortality		1.00 (reference)	1.40 (0.98-2.00)	1.46 (0.62-3.44)
Cardiovascular disease mortality		1.00 (reference)	1.53 (0.76-3.06)	1.23 (0.33-4.54)
Ischemic heart disease mortality		1.00 (reference)	2.31 (0.95-5.61)	1.08 (0.14-8.52)

Abbreviation: NHANES, National Health and Nutrition Examination Survey.

[a] Adjusted for age, sex, race and Hispanic origin, 8 cardiovascular health metrics, alcohol use, marital status, education, poverty, chronic kidney disease, family history of cardiovascular disease, and antidepressant use.

© 2023 Zhang Z et al. *JAMA Network Open.*

eTable 3. Hazard ratios and 95% CI of depressive symptoms for death from all-cause, cardiovascular disease, and ischemic heart disease—excluding participants who died within 1 year of follow-up in US adults, NHANES 2005-2018 Linked Mortality File Through 2019

	No depressive symptoms	Mild depressive symptoms	Moderate-to-severe depressive symptoms
Number of participants	18 255	3529	1783
Person-years of follow-up	142 541	26 767	13 504
All-cause mortality			
Number of deaths	1033	233	131
Weighted mortality rate (per 1000 person-years) and 95% CI	5.22 (4.74-5.67)	7.64 (6.16-9.03)	9.12 (7.06-10.97)
Model 1[a]	1.00 (reference)	1.72 (1.33-2.24)	2.48 (1.88-3.27)
Model 2[b]	1.00 (reference)	1.43 (1.10-1.86)	1.77 (1.33-2.35)
Model 3[c]	1.00 (reference)	1.36 (1.05-1.76)	1.62 (1.21-2.17)
Cardiovascular disease mortality			
Number of deaths	330	86	47
Weighted mortality rate (per 1000 person-years) and 95% CI	1.46 (1.29-1.62)	2.43 (1.82-3.00)	2.70 (1.64-3.66)
Model 1[a]	1.00 (reference)	2.03 (1.50-2.74)	2.98 (1.98-4.48)
Model 2[b]	1.00 (reference)	1.65 (1.24-2.21)	2.05 (1.39-3.04)
Model 3[c]	1.00 (reference)	1.57 (1.17-2.12)	1.90 (1.27-2.84)
Ischemic heart disease mortality			
Number of deaths	129	33	24
Weighted mortality rate (per 1000 person-years) and 95% CI	0.65 (0.53-0.77)	0.69 (0.42-0.95)	1.61 (0.63-2.49)
Model 1[a]	1.00 (reference)	1.28 (0.75-2.17)	3.84 (2.01-7.33)
Model 2[b]	1.00 (reference)	1.05 (0.62-1.78)	2.76 (1.49-5.11)
Model 3[c]	1.00 (reference)	0.97 (0.57-1.67)	2.44 (1.30-4.55)

Abbreviation: CI, confidence intervals; NHANES, National Health and Nutrition Examination Survey.

[a] Model 1: Age, sex, race and Hispanic origin adjusted.

[b] Model 2: In addition to model 1, additionally adjusted for marital status, education, poverty, chronic kidney disease, family history of cardiovascular disease, and antidepressant use.

[c] Model 3: In addition to model 2, additionally adjusted for the 8 cardiovascular health metrics developed by the American Heart Association and for alcohol use.

© 2023 Zhang Z et al. *JAMA Network Open.*

eTable 4. Hazard ratios and 95% CI of depressive symptoms for death from all-cause, cardiovascular disease, and ischemic heart disease—including participants with history of cardiovascular disease and cancer, in US adults, NHANES 2005-2018 Linked Mortality File Through 2019

	No depressive symptoms	Mild depressive symptoms	Moderate-to-severe depressive symptoms
Number of participants	21,899	4470	2505
Person-years of follow-up	166 786	32 525	18 402
All-cause mortality			
Number of deaths	2011	484	295
Weighted mortality rate (per 1000 person-years) and 95% CI	8.64 (8.06-9.20)	12.24 (10.67-13.73)	13.46 (11.32-15.41)
Model 1[a]	1.00 (reference)	1.73 (1.48-2.02)	2.42 (1.99-2.93)
Model 2[b]	1.00 (reference)	1.35 (1.15-1.60)	1.60 (1.30-1.96)
Model 3[c]	1.00 (reference)	1.31 (1.11-1.54)	1.45 (1.17-1.81)
Cardiovascular disease mortality			
Number of deaths	682	176	93
Weighted mortality rate (per 1000 person-years) and 95% CI	2.79 (2.53-3.04)	4.03 (3.35-4.67)	3.88 (2.73-4.93)
Model 1[a]	1.00 (reference)	1.80 (1.48-2.18)	2.40 (1.75-3.30)
Model 2[b]	1.00 (reference)	1.36 (1.12-1.65)	1.50 (1.08-2.10)
Model 3[c]	1.00 (reference)	1.32 (1.08-1.61)	1.36 (0.96-1.91)
Ischemic heart disease mortality			
Number of deaths	289	84	48
Weighted mortality rate (per 1000 person-years) and 95% CI	1.28 (1.09-1.47)	1.78 (1.31-2.22)	2.22 (1.32-3.03)
Model 1[a]	1.00 (reference)	1.75 (1.28-2.39)	2.97 (1.96-4.51)
Model 2[b]	1.00 (reference)	1.31 (0.96-1.77)	1.84 (1.14-2.97)
Model 3[c]	1.00 (reference)	1.24 (0.91-1.70)	1.59 (0.97-2.59)

Abbreviation: CI, confidence intervals; NHANES, National Health and Nutrition Examination Survey.

[a] Model 1: Age, sex, race and Hispanic origin adjusted.

[b] Model 2: In addition to model 1, additionally adjusted for marital status, education, poverty, history of cardiovascular disease, cancer, chronic kidney disease, family history of cardiovascular disease, and antidepressant use.

[c] Model 3: In addition to model 2, additionally adjusted for the 8 cardiovascular health metrics developed by the American Heart Association and for alcohol use.

© 2023 Zhang Z et al. *JAMA Network Open.*

eTable 5. Characteristics across depressive symptoms after multiple imputation among participants eligible for mortality analyses (n=27 566), NHANES 2005-2018

	All (N=27 566)	No depressive symptoms (n = 21 282)	Mild depressive symptoms (n = 4168)	Moderate-to-severe depressive symptoms (n = 2116)	*P* value
	**Sociodemographic and Clinical Characteristics**				
**Age, mean (se)**	44.6 (0.22)	44.9 (0.23)	43.6 (0.42)	43.5 (0.47)	<0.001
**Sex, N (%)[a]**					<0.001
Men	13 593 (49.3)	11 055 (51.5)	1763 (43.2)	775 (37.9)	
Women	13 973 (50.7)	10 227 (48.5)	2405 (56.8)	1341 (62.1)	
**Race and Hispanic origin, N (%)[a]**					0.002
Hispanic	7512 (15.1)	5717 (14.9)	1159 (16.5)	636 (18.0)	
Non-Hispanic Black	6123 (11.8)	4701 (11.4)	949 (12.7)	474 (14.0)	
Non-Hispanic White	10 822 (64.8)	8324 (65.6)	1649 (62.6)	849 (60.9)	
Other[b]	3109 (8.0)	2540 (8.0)	411 (8.2)	158 (7.1)	
**Marital status, N (%)[a]**					<0.001
Never married	5619 (21.1)	4126 (20.1)	978 (23.9)	515 (26.2)	
Married/living with partner	16 565 (62.3)	13 389 (65.2)	2214 (55.2)	963 (45.8)	
Divorced, separated, or widowed	5382 (16.6)	3767 (14.7)	977 (20.8)	638 (28.0)	
**Poverty index ratio[c], N (%)[a]**					<0.001
<1.30	8450 (21.8)	5826 (18.6)	1555 (28.1)	1068 (41.4)	
1.30-3.49	10 438 (35.2)	8087 (34.4)	1631 (39.5)	720 (35.9)	
≥3.5	8678 (42.9)	7369 (46.9)	981 (32.5)	328 (22.7)	
**Education level, N (%)[a]**					<0.001
<12 years	6618 (15.3)	4758 (13.9)	1137 (18.9)	723 (23.7)	
12 years	6310 (23.4)	4781 (22.6)	1021 (26.2)	508 (27.2)	
>12 years	14 638 (61.2)	11 743 (63.6)	2010 (55.0)	885 (49.1)	
**Alcohol use, N (%)[a]**					<0.001
No use	15 526 (49.6)	11852 (48.7)	2358 (50.1)	1316 (57.8)	
Moderate use	9588 (39.6)	7683 (41.2)	1350 (36.0)	555 (28.7)	
Heavy use	2452 (10.9)	1746 (10.1)	461 (13.9)	244 (13.5)	
**Insurance status, N (%)[a]**					<0.001
Uninsured	6671 (19.7)	4873 (18.2)	1162 (24.3)	636 (27.4)	
Insured	20 895 (80.3)	16 409 (81.8)	3006 (75.7)	1479 (72.6)	

© 2023 Zhang Z et al. *JAMA Network Open.*

**Family history of cardiovascular disease, N (%)[a]**					<0.001
Yes	2980 (11.6)	2016 (10.3)	586 (15.0)	378 (18.7)	
No	24 586 (88.4)	19 266 (89.7)	3582 (85.0)	1738 (81.3)	
**Chronic kidney disease, N (%)[a]**					0.292
Yes	3982 (11.5)	2957 (11.2)	661 (12.6)	364 (12.5)	
No	23 584 (88.5)	18326 (88.8)	3507 (77.4)	1751 (87.5)	
**Antidepressant use, N (%)[a]**					<0.001
Yes	2491 (11.4)	1227 (7.8)	660 (20.0)	604 (32.5)	
No	25 075 (88.6)	20 055 (92.2)	3508 (80.0)	1512 (67.5)	
**LE8 individual scores, mean (SE)**					
Body mass index	60.7 (0.44)	62.1 (0.48)	56.2 (0.82)	54.6 (1.18)	<0.001
Nicotine exposure	70.9 (0.48)	74.2 (0.46)	63.1 (1.03)	52.2 (1.71)	<0.001
Physical activity	52.2 (0.80)	55.3 (0.80)	45.3 (1.26)	33.4 (1.53)	<0.001
Hypertension	70.5 (0.35)	70.7 (0.39)	69.9 (0.77)	69.6 (1.14)	0.962
Diabetes	80.4 (0.27)	80.8 (0.32)	79.7 (0.60)	78.0 (0.79)	0.233
Non-HDL cholesterol	64.6 (0.36)	65.0 (0.38)	63.0 (0.80)	63.8 (1.15)	0.748
Diet	39.2 (0.48)	40.8 (0.51)	35.8 (0.82)	30.1 (0.93)	<0.001
Sleep health	83.1 (0.26)	85.3 (0.24)	78.0 (0.66)	69.9 (1.17)	<0.001
**LE8 summary scores, mean (SE)**	65.2 (0.29)	66.8 (0.29)	61.4 (0.44)	56.5 (0.51)	<0.001
**LE8 levels, N (%)[a]**					<0.001
Low	5415 (16.6)	3526 (13.4)	1129 (23.8)	760 (32.8)	
Moderate	17 861 (64.3)	14 031 (65.1)	2606 (62.4)	1224 (59.8)	
High	4290 (19.1)	3726 (21.5)	433 (13.8)	132 (7.4)	

Abbreviations: CVH, cardiovascular health; HDL, high density lipoprotein; NHANES, National Health and Nutrition Examination Survey; se, standard error.

[a] Unweighted N, and weighted %.

[b] Other race includes non-Hispanic Asian, non-Hispanic Native Hawaiian or other Pacific Islander, non-Hispanic American Indian or Alaska Native, or multi-racial.

[c] Poverty-to-income ratio is the ratio of family income to the Department of Health and Human Services poverty measure.

© 2023 Zhang Z et al. *JAMA Network Open.*

eTable 6. Unadjusted and adjusted hazard ratios and 95% CI of depressive symptoms for death from all cause, cardiovascular disease and ischemic heart disease after multiple imputation among participants eligible for mortality analyses (n=27 566), NHANES 2005-2018 Linked Mortality File Through 2019

	**No depressive symptoms**	**Mild depressive symptoms**	**Moderate-to-severe depressive symptoms**
Number of participants	21 282	4168	2116
Person-years of follow-up	163 397	31 269	15 547
All-cause mortality			
Number of deaths	1395	317	181
Weighted mortality rate (per 1000 person-years) and 95% CI	6.09 (6.09-6.10)	8.10 (7.86-8.33)	10.00 (8.92-10.98)
Model 1[a]	1.00 (reference)	1.61 (1.29-2.02)	2.37 (1.87-3.00)
Model 2[b]	1.00 (reference)	1.34 (1.07-1.69)	1.62 (1.25-2.10)
Model 3[c]	1.00 (reference)	1.28 (1.02-1.60)	1.49 (1.14-1.93)
Cardiovascular disease mortality			
Number of deaths	439	112	58
Weighted mortality rate (per 1000 person-years) and 95% CI	1.74 (1.73-1.74)	2.44 (2.36-2.51)	2.63 (2.21-3.01)
Model 1[a]	1.00 (reference)	1.73 (1.31-2.30)	2.41 (1.63-3.55)
Model 2[b]	1.00 (reference)	1.46 (1.10-1.94)	1.65 (1.09-2.49)
Model 3[c]	1.00 (reference)	1.39 (1.03-1.86)	1.52 (1.01-2.31)
Ischemic heart disease mortality			
Number of deaths	176	44	28
Weighted mortality rate (per 1000 person-years) and 95% CI	0.77 (0.77-0.78)	0.79 (0.75-0.82)	1.47 (1.41-1.52)
Model 1[a]	1.00 (reference)	1.28 (0.81-2.01)	2.95 (1.65-5.28)
Model 2[b]	1.00 (reference)	1.08 (0.69-1.71)	2.09 (1.16-3.74)
Model 3[c]	1.00 (reference)	1.00 (0.62-1.61)	1.86 (1.03-3.39)

Abbreviation: CI, confidence intervals; NHANES, National Health and Nutrition Examination Survey.

[a] Model 1: Age, sex, race and Hispanic origin adjusted.

[b] Model 2: In addition to model 1, additionally adjusted for marital status, education, poverty, chronic kidney disease, family history of cardiovascular disease, and antidepressant use.

[c] Model 3: In addition to model 2, additionally adjusted for the 8 cardiovascular health metrics developed by the American Heart Association and for alcohol use.

© 2023 Zhang Z et al. *JAMA Network Open.*
