# Supplementary material for: Depressive Symptoms and Mortality Among US Adults
Source: JAMA Netw Open. 2023 Oct 9;6(10):e2337011. doi: 10.1001/jamanetworkopen.2023.37011 (PMC10562940; doi:10.1001/jamanetworkopen.2023.37011)
Supplement: Supplement 2. — Data Sharing Statement [file jamanetwopen-e2337011-s002.pdf]

Data Sharing Statement

Data
Data available: No

Additional Information
Explanation for why data not available: Cardiovascular disease and ischemic disease mortality data can only be accessed at the Restricted Data Center, National Center for Health Statistics. All other data and data dictionary defining each field in the analyses will be available.
